# Influence of weight status on 24-hour urine composition in adults without urolithiasis: A nationwide study based on a Chinese Han population

**DOI:** 10.1371/journal.pone.0184655

**Published:** 2017-09-08

**Authors:** Tuo Deng, Zanlin Mai, Chao Cai, Xiaolu Duan, Wei Zhu, Tao Zhang, Wenqi Wu, Guohua Zeng

**Affiliations:** 1 Department of Urology, Minimally Invasive Surgery Center, The First Affiliated Hospital of Guangzhou Medical University, Guangzhou, China; 2 Guangzhou Institute of Urology, Guangzhou, China; 3 Guangdong Key Laboratory of Urology, Guangzhou, China; Sun Yat-sen University, CHINA

## Abstract

**Objectives:**

This study sought to explore the influence of different body weight statuses on 24-hour urine compositions in adults without urolithiasis based on a nationwide study of a Chinese Han population.

**Material and methods:**

Twenty-four-hour urine samples from 584 Chinese Han adults without urolithiasis in six cities were analyzed. The participants were divided into four body weight status types according to their body mass indices (BMIs) according to WHO guidelines. The baseline characteristics and 24-hour urine compositions of the standard weight group were compared with those of the underweight, overweight and obese groups. The influences of different body weight statuses on the 24-hour urine compositions were explored using univariate and multivariate logistic regressions.

**Results:**

The numbers of participants in the underweight, standard weight, overweight and obese status groups were 24, 376, 149 and 35, respectively. The overweight and obese groups suffered significantly higher risks of hypertension and diabetes mellitus than the standard weight group. In the univariate analyses, compared with the standard weight group, the overweight group had significantly higher levels of urine citrate (mean difference [MD] = 0.51 mmol, 95% confidence interval [CI]: 0.15–0.87, *P* = 0.001), potassium (MD = 6.63 mmol, 95% CI: 1.13–12.14, *P* = 0.01) and magnesium (MD = 0.38 mmol, 95% CI: 0.08–0.69, *P* = 0.014). Significant increases in urine citrate (MD = 0.85 mmol, 95% CI: 0.01–1.68, *P* = 0.046), magnesium (MD = 0.69 mmol, 95% CI: 0.13–1.25, *P* = 0.016) and phosphate (MD = 2.28 mmol, 95% CI: 0.03–4.54, *P* = 0.047) were found in the obese group. No significant differences were detected between the standard weight and underweight groups. In the multivariate logistic regression analyses, we only observed significantly higher levels of urine potassium (odds ratio [OR] = 1.02, 95% CI: 1.00–1.04, *P* = 0.03) in the overweight group and phosphate (OR = 1.32, 95% CI: 1.05–1.66, *P* = 0.018) in the obese group when compared with the standard weight group.

**Conclusions:**

Nonstone-forming adults with overweight or obese statuses were at higher risks of hypertension and diabetes mellitus. Obese nonstone-formers might have a greater risk of urinary stone formation due to increased urinary phosphate excretion. Additionally, underweight status had no influence on 24-hour urine composition.

## Introduction

Overweight and obese statuses are common health problems in developed countries and some developing countries such as China. Weight gain has also been proven to associated with increased risks of several diseases, including hypertension, diabetes mellitus, coronary heart disease, etc. Additionally, previous studies have researched the relationship between body mass index (BMI) and the risk of urolithiasis in recent decades [1–5].

Urolithiasis is among the most common diseases of the urinary system. China is one of three main urinary stone epidemic areas in the world in which the incidence has been reported to be as high as 1% to 5% [6]. Urolithiasis is also a heavy burden disease due to its high recurrence rate [7, 8]. According to research into the mechanism of this disease, urinary stone formation is associated with multiple factors including heredity, diet, environment and metabolism.

Body weight status, as categorized according to the BMI, reflects the condition of the body’s metabolism, which is expected to be related to urinary stone formation. Accordingly, the metabolic evaluation of the 24-hour urine composition, which is representative of the ionic microenvironment of the collection system, has now become an essential and important auxiliary examination for patients with urolithiasis. Therefore, previous studies have investigated the influences of overweight and obese statuses on the 24-hour urine compositions of patients with urolithiasis [5, 9–11]. However, no study yet exists that has focused on the changes in and comparisons of 24-hour urine compositions among nonstone-forming populations with different body weight statuses. Our study aimed to explore the influences of different body weight statuses on the 24-hour urine compositions of adults without urolithiasis based on a nationwide epidemiological study in the Chinese Han population.

## Material and methods

### Study population

This study was part of a nationwide epidemiological study of urolithiasis among adults aged 18 and older in China from May 2013 to July 2014. A total of 584 Chinese Han adults without urolithiasis from six cities from six provinces were included in this study. The six cities were as follows: Shanghai, Chongqing, Harbin, Shaoyang, Lanzhou, and Changzhi. All participants were non-incentivized volunteers. Our study was approved by the Ethics Committee of the First Affiliated Hospital of Guangzhou Medical University, China, and written informed consent was obtained from every participant.

Subjects with following features were excluded: incomplete urine samples (urine creatinine < 600 μg/24 h); serum creatinine > 133 μmol/L; urinary stones; urinary tract infection; gout; hyperthyroidism; malignancy; enterectomy history; and a history of the use of thiazide, allopurinol, vitamin supplements, potassium citrate or calcium supplements in last two weeks. All eligible participants with a free diet received a urinary tract ultrasonographic examination and a questionnaire to provide their basic information (e.g., age, gender, health history). Blood and urine samples from participants were analyzed by skillful experts using standardized protocols. The estimated creatinine clearance rate (eCCr) was calculated according to the Cockcroft-Gault equation.

### Collection and analysis of 24-hour urine samples

Detailed methods of the collection and analyses of the 24-hour urine samples have been described in our previous study [12]. The main measurements of the 24-hour urine analysis included the urine volume, pH value and concentrations of urine sodium, potassium, chloride, calcium, phosphate, uric acid, magnesium, oxalate and citrate. Additionally, the ion activities of the products of calcium oxalate and calcium phosphate, expressed in terms of the AP (CaOx) index_s_ and the AP (CaP) index_s_, were also calculated according to the formulas below [13]. The 24-hour urine analyses were performed in the Guangdong Key Laboratory of Urology according to standardized protocols.

$$AP(CaOx){index}_{s}=\frac{1.9*{Calcium}^{0.84}*Oxalate}{{Citrate}^{0.22}*{Magnesium}^{0.12}*1.5^{1.03}}$$

$$AP(CaP){index}_{s}=\frac{2.7*10^{-3}*{Calcium}^{1.07}*{Phospate}^{0.70}*{\left(7.0-4.5\right)}^{6.8}}{{Citrate}^{0.20}*1.5^{1.31}}$$

### Weight status category

The heights and weights of all participants were measured by trained researchers during their personal face-to-face interviews, and the BMIs were then calculated according as the body weight in kilograms/(body height in meters)^2^. The body weight statuses in this study were categorized according to BMI based on the WHO guidelines: underweight status (BMI < 18.5 kg/m^2^), standard weight status (18.5–24.9 kg/m^2^), overweight status (25–29.9 kg/m^2^), and obese status (BMI ≥ 30 kg/m^2^) [14].

### Statistical analysis

The baseline characteristics of the standard weight group were compared with those of the underweight, overweight and obese groups. Student’s t-tests were used for continuous variables, and chi-square tests were used for categorical variables. Univariate comparisons of the urinary components of the standard weight group with those of the underweight, overweight and obese groups were conducted with Student’s t-tests. A multivariate logistic regression was performed to adjust for possible confounders, including age, gender, hypertension, diabetes mellitus, hyperuricemia, eCCr, serum total cholesterol, triglyceride, low-density lipoprotein (LDL) and high-density lipoprotein (HDL). Moreover, comparisons between the overweight and obese groups were also conducted. A two-sided *P* value < 0.05 was considered significant for the results obtained in this study. All statistical analyses were conducted with the SPSS 13.0 software.

## Results

A total of 584 adults without urolithiasis were included in this study. The numbers of participants in the underweight, standard weight, overweight and obese status groups were 24, 376, 149 and 35, respectively. The baseline characteristics of all participants in the 4 groups are listed in Table 1. Compared with the standard weight group, no significant differences were found in age or gender in the underweight, overweight or obese groups. However, eCCr increased significantly with weight gain. The overweight and obese groups had significant higher risks of hypertension and diabetes mellitus and a lower level of serum HDL. Higher serum triglyceride levels were also observed in the overweight group. The comparisons of the baseline characteristics between the overweight and obese groups are presented in Table 2. Significantly higher risks of hypertension and diabetes mellitus were discovered in the obese group compared with the overweight group.

**Table 1:** Baseline characteristics of all the participants in the four weight status groups.

*Characteristic*	*Standard weight adults*	*Underweight adults*	P* *Value*	*Overweight adults*	P* *Value*	*Obese adults*	P* *Value*
	*(n = 376)*	*(n = 24)*		*(n = 149)*		*(n = 35)*	
Age (year)	48.86 ± 16.25	53.96 ± 19.28	0.746	52.14 ± 14.25	0.13	50.03 ± 12.84	0.996
BMI (kg/m2)	22.10 ± 1.73	17.39 ± 0.90	**<0.001**	26.70 ± 1.32	**<0.001**	32.81 ± 4.53	**<0.001**
Gender, Male (n [%])	188 (50.0)	12 (50.0)	1	64 (43.0)	0.145	14 (40.0)	0.258
Hypertension (n [%])	78 (20.7)	4 (16.7)	0.632	47 (31.5)	**0.009**	19 (54.3)	**<0.001**
Diabetes mellitus (n [%])	59 (15.7)	6 (25.0)	0.231	37 (24.8)	**0.015**	20 (57.1)	**<0.001**
Hyperuricemia (n [%])	26 (6.9)	2 (8.3)	0.792	13 (8.7)	0.476	1 (2.9)	0.355
Hemoglobin (g/L)	139.58 ± 17.05	138.38 ± 14.15	0.735	138.95 ± 17.10	0.702	143.40 ± 15.97	0.201
Serum creatinine (μmol/L)	73.66 ± 17.14	73.06 ± 20.44	0.867	71.36 ± 16.52	0.164	68.17 ± 16.28	0.069
eCCr (ml/min)	85.02 ± 26.95	64.42 ± 18.63	**<0.001**	99.07 ± 28.60	**<0.001**	124.45 ± 43.27	**<0.001**
Serum calcium (mmol/L)	2.39 ± 0.12	2.35 ± 0.29	0.983	2.38 ± 0.15	0.998	2.39 ± 0.13	1
Serum uric acid (μmol/L)	292.28 ± 83.13	277.82 ± 94.93	0.41	299.13 ± 83.02	0.396	297.82 ± 78.24	0.707
Total cholesterol (mmol/L)	4.62 ± 1.11	4.89 ± 0.96	0.238	4.77 ± 1.01	0.138	4.62 ± 1.10	0.992
Triglyceride (mmol/L)	1.23 ± 1.54	1.17 ± 1.37	0.849	1.65 ± 1.36	**0.003**	1.46 ± 0.92	0.363
Low-density lipoprotein (mmol/L)	2.69 ± 0.79	2.40 ± 0.70	0.081	2.76 ± 0.78	0.407	2.83 ± 1.04	0.322
High-density lipoprotein (mmol/L)	1.37 ± 0.36	1.54 ± 0.42	0.336	1.21 ± 0.27	**<0.001**	1.20 ± 0.27	**0.006**

^*^The *P* values were calculated based on comparisons with the standard weight adults.

BMI: body mass index; eCCr: estimated creatinine clearance rate.

The bold numbers indicate *P* values < 0.05.

**Table 2:** Comparisons of the baseline characteristics between the overweight and obese adults without urolithiasis.

*Characteristic*	*Overweight adults*	*Obese adults*	P *Value*
	*(n = 149)*	*(n = 35)*	
Age (year)	52.14 ± 14.25	50.03 ± 12.84	0.947
BMI (kg/m2)	26.70 ± 1.32	32.81 ± 4.53	**<0.001**
Gender, Male (n [%])	64 (43.0)	14 (40.0)	0.751
Hypertension (n [%])	47 (31.5)	19 (54.3)	**0.012**
Diabetes mellitus (n [%])	37 (24.8)	20 (57.1)	**<0.001**
Hyperuricemia (n [%])	13 (8.7)	1 (2.9)	0.24
Hemoglobin (g/L)	138.95 ± 17.10	143.40 ± 15.97	0.162
Serum creatinine (μmol/L)	71.36 ± 16.52	68.17 ± 16.28	0.321
eCCr (ml/min)	99.07 ± 28.60	124.45 ± 43.27	**0.012**
Serum calcium (mmol/L)	2.38 ± 0.15	2.39 ± 0.13	0.999
Serum uric acid (μmol/L)	299.13 ± 83.02	297.82 ± 78.24	0.933
Total cholesterol (mmol/L)	4.77 ± 1.01	4.62 ± 1.10	0.45
Triglyceride (mmol/L)	1.65 ± 1.36	1.46 ± 0.92	0.492
Low-density lipoprotein (mmol/L)	2.76 ± 0.78	2.83 ± 1.04	0.614
High-density lipoprotein (mmol/L)	1.21 ± 0.27	1.20 ± 0.27	1

BMI: body mass index; eCCr: estimated creatinine clearance rate.

The bold numbers indicate *P* values < 0.05.

The univariate comparisons of the urinary components of the standard weight group with those of the underweight, overweight, obese groups are presented in in Table 3. No significant differences were found in the 24-hour urine compositions between the standard weight and underweight groups. However, the overweight group exhibited significantly higher levels of urine citrate (mean difference [MD] = 0.51 mmol, 95% confidence interval [CI]: 0.15–0.87, *P* = 0.001), potassium (MD = 6.63 mmol, 95% CI: 1.13–12.14, *P* = 0.01) and magnesium (MD = 0.38 mmol, 95% CI: 0.08–0.69, *P* = 0.014) compared with the standard weight group. Significant increases in urine citrate (MD = 0.85 mmol, 95% CI: 0.01–1.68, *P* = 0.046), magnesium (MD = 0.69 mmol, 95% CI: 0.13–1.25, *P* = 0.016) and phosphate (MD = 2.28 mmol, 95% CI: 0.03–4.54, *P* = 0.047) were also detected in the obese group compared with the standard weight group. The univariate comparisons of the 24-hour urine compositions between the overweight and obese groups are detailed in Table 4. No significant differences were observed in the urinary components between the overweight and obese groups.

**Table 3:** Univariate comparisons of the 24-hour urine compositions between the standard weight adults and the underweight, overweight, and obese adults without urolithiasis.

*Characteristic*	*Standard weight adults*	*Underweight adults*	P* *Value*	*Overweight adults*	P* *Value*	*Obese adults*	P* *Value*
	*(n = 376)*	*(n = 24)*		*(n = 149)*		*(n = 35)*	
Calcium (mmol)	4.15 ± 2.14	3.97 ± 2.03	0.691	4.25 ± 2.36	0.646	4.20 ± 2.39	0.907
Oxalate (mmol)	0.26 ± 0.15	0.22 ± 0.09	0.418	0.27 ± 0.15	0.977	0.27 ± 0.14	0.999
Citrate (mmol)	1.98 ± 1.28	1.78 ± 1.14	0.947	2.49 ± 1.46	**0.001**	2.83 ± 1.74	**0.046**
Uric acid (mmol)	3.11 ± 1.13	3.03 ± 0.85	0.739	3.19 ± 1.12	0.428	3.31 ± 1.15	0.305
Sodium (mmol)	180.53 ± 164.17	207.93 ± 259.52	0.382	194.79 ± 82.34	0.322	192.66 ± 70.77	0.645
Potassium (mmol)	40.91 ± 16.46	41.41 ± 13.83	1	47.54 ± 23.09	**0.01**	45.66 ± 19.62	0.663
Magnesium (mmol)	3.50 ± 1.55	3.34 ± 1.42	0.627	3.89 ± 1.75	**0.014**	4.19 ± 1.83	**0.016**
Phosphate (mmol)	16.84 ± 6.69	16.17 ± 5.11	0.628	17.31 ± 6.23	0.452	19.12 ± 6.35	**0.047**
Chloride (mmol)	177.36 ± 167.60	205.68 ± 269.19	0.377	189.25 ± 83.73	0.42	186.95 ± 74.30	0.722
Creatinine (mmol)	9.93 ± 3.41	9.46 ± 2.71	0.481	9.95 ± 2.79	0.969	10.46 ± 2.66	0.348
Volume (ml)	1445.09 ± 611.10	1296.25 ± 425.93	0.259	1522.89 ± 672.94	0.199	1450.86 ± 675.57	0.958
pH	5.97 ± 0.71	5.94 ± 0.54	0.824	5.88 ± 0.62	0.172	6.00 ± 0.76	0.8
AP (CaOx) Index_s_	0.81 ± 0.58	0.70 ± 0.40	0.387	0.82 ± 0.70	0.826	0.77 ± 0.50	0.69
AP (CaP) Index_s_	25.44 ± 19.25	24.64 ± 16.17	0.84	24.85 ± 18.09	0.748	25.22 ± 19.46	0.947

^*^The *P* values were calculated based on comparisons with the standard weight adults.

AP: the ion activity products.

The bold numbers indicate *P* values < 0.05.

**Table 4:** Univariate comparisons of the 24-hour urine compositions between the overweight and obese adults without urolithiasis.

*Characteristic*	*Overweight adults*	*Obese adults*	P *Value*
	*(n = 149)*	*(n = 35)*	
Calcium (mmol)	4.25 ± 2.36	4.20 ± 2.39	0.899
Oxalate (mmol)	0.27 ± 0.15	0.27 ± 0.14	1
Citrate (mmol)	2.49 ± 1.46	2.83 ± 1.74	0.871
Uric acid (mmol)	3.19 ± 1.12	3.31 ± 1.15	0.577
Sodium (mmol)	194.79 ± 82.34	192.66 ± 70.77	0.939
Potassium (mmol)	47.54 ± 23.09	45.66 ± 19.62	0.997
Magnesium (mmol)	3.89 ± 1.75	4.19 ± 1.83	0.315
Phosphate (mmol)	17.31 ± 6.23	19.12 ± 6.35	0.139
Chloride (mmol)	189.25 ± 83.73	186.95 ± 74.30	0.936
Creatinine (mmol)	9.95 ± 2.79	10.46 ± 2.66	0.388
Volume (ml)	1522.89 ± 672.94	1450.86 ± 675.57	0.54
pH	5.88 ± 0.62	6.00 ± 0.76	0.346
AP (CaOx) Index_s_	0.82 ± 0.70	0.77 ± 0.50	0.625
AP (CaP) Index_s_	24.85 ± 18.09	25.22 ± 19.46	0.918

AP: the ion activity products.

Multivariate logistic regression analyses were conducted between the standard weight group and the overweight and obese groups (Table 5). After adjustments for age, gender, hypertension, diabetes mellitus, hyperuricemia, eCCr, serum total cholesterol, triglyceride, LDL and HDL, we observed significantly higher levels of urine potassium (odds ratio [OR] = 1.02, 95% CI: 1.00–1.04, *P* = 0.03) in the overweight group and phosphate (OR = 1.32, 95% CI: 1.05–1.66, *P* = 0.018) in the obese group compared with the standard weight group.

**Table 5:** Results of the multivariate logistic regression analysis of the standard weight adults and the overweight and obese adults without urolithiasis.

*Characteristic*	*Standard weight* vs. *Overweight*		*Standard weight* vs. *Obese*	
	*OR (95% CI)*	P *Value*	*OR (95% CI)*	P *Value*
Calcium (mmol)	0.95 (0.60, 1.51)	0.823	1.37 (0.47, 4.00)	0.567
Oxalate (mmol)	0.24 (0.00, 28.10)	0.558	0.113 (0.00, 16271.13)	0.719
Citrate (mmol)	1.12 (0.83, 1.50)	0.466	1.49 (0.82, 2.71)	0.195
Uric acid (mmol)	0.71 (0.49, 1.04)	0.076	0.60 (0.26, 1.37)	0.225
Sodium (mmol)	1.02 (1.00, 1.03)	0.07	0.99 (0.95, 1.02)	0.446
Potassium (mmol)	**1.02 (1.00, 1.04)**	**0.03**	0.96 (0.91, 1.01)	0.093
Magnesium (mmol)	1.02 (0.83, 1.25)	0.857	1.24 (0.78, 1.98)	0.364
Phosphate (mmol)	0.99 (0.90, 1.08)	0.789	**1.32 (1.05, 1.66)**	**0.018**
Chloride (mmol)	0.98 (0.97, 1.00)	0.051	1.01 (0.98, 1.05)	0.514
Creatinine (mmol)	1.06 (0.91, 1.23)	0.482	1.35 (0.93, 1.95)	0.114
Volume (ml)	1	0.889	1	0.227
pH	0.72 (0.47, 1.08)	0.114	1.14 (0.51, 2.57)	0.747
AP (CaOx) Index_s_	1.66 (0.47, 5.90)	0.434	2.10 (0.70, 63.08)	0.669
AP (CaP) Index_s_	0.99 (0.92, 1.05)	0.691	0.88 (0.76, 1.02)	0.087

AP: the ion activity products; CI: confidence interval; OR: odds ratio.

The bold numbers indicate *P* values < 0.05.

## Discussion

Five hundred eighty-four nonstone-forming adults from six different cities in China were included in our study. We divided all the participants into four groups according to their body weight statuses. Based on comparisons of the baseline characteristics, we concluded that the participants in the overweight and obese status groups were at significantly higher risks of hypertension and diabetes mellitus, had lower serum HDL levels, and had greater eCCr levels than those in the standard weight status groups. Univariate analyses of the urinary components indicated that the participants in the overweight status group had significantly higher levels of urine citrate, potassium and magnesium. Moreover, urine citrate, magnesium and phosphate were significantly elevated in the obese group. Comparisons between the underweight and standard weight groups revealed no significant differences, and there were also no significant differences between the overweight and obese groups. A multivariate analysis indicated that only urine potassium in the overweight group and phosphate in the obese group were significantly increased compared with the standard weight group after adjustments for all possible confounding factors.

Greater BMI has already been proven to be associated with increased risks of hypertension and diabetes mellitus [15–17]. In our study, we also drew the conclusion that the participants in the overweight and obese groups were at significantly increased risks of hypertension and diabetes mellitus compared with the standard weight adults. Previous studies have investigated the influences of hypertension and diabetes mellitus on 24-hour urine compositions. Hartman et al [18] found that patients with nephrolithiasis suffering from hypertension have lower levels of urine citrate than those without hypertension based on a retrospective study that included 1115 patients. In the study by Eisner et al [19, 20], only a significant increase in urine calcium was observed in hypertensive stone formers, and higher urinary oxalate and lower urine pH were found in stone-forming patients with type II diabetes mellitus compared with non-diabetic patients. According to these findings, hypertension and diabetes mellitus appear to be significant risk factors for urinary stones. Therefore, overweight and obese statuses, which can increase the risk of hypertension and diabetes mellitus, also probably tend to increase the risk of urolithiasis.

In our multivariate analysis, increased urine potassium was observed in the overweight adults compared with †he standard weight group after adjustments for possible confounding factors that included age, gender, hypertension, diabetes mellitus, etc. Taylor et al [21] and Eisner et al [22] also discovered that increased BMI is associated with a significant increase in urinary potassium excretion in stone formers based on a univariate analysis, although a subsequent multivariate analysis demonstrated no significant difference. Furthermore, previous studies have indicated that increased urinary potassium excretion is correlated with a decreased risk of stone formation [23] and growth [24]. Potassium depletion has also been suggested to elevate the risk of stone formation through enhanced supersaturation [25, 26]. Consequently, elevating the urine potassium level in overweight nonstone-formers seemed to be a protective factor against urolithiasis. However, it is notable that a similar increase in urine potassium was not observed in the obese adults, and the level of urine potassium exhibited no significant difference between the overweight and obese groups. Therefore, we consider that increased urinary potassium excretion in the overweight group might have been caused by some non-adjustable confounders, such as diet, because it was impossible to apply a standard and controlled diet to all the participants in our study.

Compared with the standard weight group, the non-stone formers in the obese status group exhibited a significantly higher level of urine phosphate in our multivariate analysis. This result is consistent with those of previous studies [1, 21, 22, 27]. Khand et al [28] reported a positive association between hypocitraturia and phosphaturia in first-time stone formers, and the high level of urine phosphate was thought to be one of the main risk factors for calcium urolithiasis. Gyawali et al [29] also observed a significantly higher urine phosphate level in stone formers than in nonstone formers, which indicates that increased urinary phosphate excretion might be a significant risk factor for urinary stones. Hence, our study found that obese adults without urolithiasis might suffer a greater risk of urinary stone formation than standard weight populations due to the relatively high urine phosphate level. However, potential confounders from the various diets could not be excluded.

Currently, few studies have investigated the influence of underweight status on 24-hour urine composition and the risk of urolithiasis. Our study demonstrated that, with the exception of eCCr, no significant differences were found in the baseline characteristics or 24-hour urine compositions between the underweight and standard weight nonstone formers. Thus, we suggest that no significant difference exists in the risk of urinary stone formation between underweight and standard weight populations. Moreover, in the comparisons between the overweight and obese groups, although no significant differences were detected in the 24-hour urine compositions, the higher risks of hypertension and diabetes mellitus might also increase the risk of urolithiasis among obese nonstone formers.

Some limitations of our study should be stated. Because our study is one part of a nationwide epidemiological study of urolithiasis, the dietary habits of the participants could not be controlled and likely differed widely due to the remote locations of some of the sample population. Different diets might have potentially influenced the 24-hour urine compositions but could not be adjusted for in the multivariate analysis. Furthermore, the climates and environments were also different across the included cities, and the numbers of underweight and obese participants in our study were relatively small. These factors may have introduced potential biases into our results.

## Conclusions

Our study concluded that nonstone-forming adults with overweight or obese statuses were at higher risks of hypertension and diabetes mellitus than standard weight adults and thus tended to be more susceptible to urolithiasis. A positive association was observed between obese status and increased urinary phosphate excretion, which indicates a greater risk of urinary stone formation in obese patients. Additionally, no significant differences were found in the 24-hour urine compositions of the underweight and standard weight nonstone-forming adults.
